# Decoding the Inflammatory Pathway in Heart Failure: The Role of Interleukins and Tumor Necrosis Factor-Alpha in Disease Severity

**DOI:** 10.3390/jcm14176092

**Published:** 2025-08-28

**Authors:** Sameh A. Ahmed, Hussein M. Ismail, Ahmed B. Alahmedi, Faisal B. Alahmadi, Abdulaziz F. Muhawish, Abed A. Alsubhi, Yazeed S. Almohammadi, Abdulrahman K. Alwusaidi, Abdullah S. Alsaedi, Tariq G. Alhazmi, Mohammed N. Busra

**Affiliations:** 1 Department of Pharmacognosy and Pharmaceutical Chemistry, College of Pharmacy, Taibah University, Al-Madinah Al-Munawarah 30001, Saudi Arabia; 2 Department of Internal Medicine, College of Medicine, Taibah University, Al-Madinah Al-Munawarah 30001, Saudi Arabia; hismail@taibahu.edu.sa (H.M.I.);; 3 Adult Cardiology, Madinah Cardiac Center, Al-Madinah Al-Munawara 42351, Saudi Arabia; aalahmedi@moh.gov.sa

**Keywords:** heart failure, inflammatory markers, left ventricular ejection fraction

## Abstract

**Background/Objectives**: Heart failure (HF) remains a major global cause of morbidity and mortality, exerting substantial health and economic burdens. Increasing evidence suggests that systemic inflammation plays a pivotal role in HF pathophysiology, with key cytokines; interleukin-1 (IL-1), interleukin-6 (IL-6), and tumor necrosis factor-alpha (TNF-α) contributing to disease progression and worsening cardiac function. This study aimed to evaluate serum levels of IL-1, IL-6, and TNF-α in patients with HF compared to control subjects, to assess differences in these inflammatory mediators between groups, and to explore their relationship with left ventricular ejection fraction (LVEF). **Methods**: A case–control study was conducted at the Madinah Cardiac Center between October 2024 and April 2025, including 61 patients diagnosed with HF and 65 age- and sex-matched controls without HF. Serum concentrations of IL-1, IL-6, and TNF-α were measured using enzyme-linked immunosorbent assay (ELISA). Clinical parameters, including LVEF and echocardiographic data, were recorded and analyzed. **Results**: Patients with HF demonstrated significantly elevated levels of IL-1 (6.77 ± 1.17 vs. 1.27 ± 0.42 pg/mL, *p* < 0.001), IL-6 (54.12 ± 4.64 vs. 9.29 ± 1.72 pg/mL, *p* < 0.001), and TNF-α (235.56 ± 18.88 vs. 67.37 ± 6.28 pg/mL, *p* < 0.001) compared to controls. Higher inflammatory marker levels were associated with reduced LVEF and more advanced New York Heart Association (NYHA) functional class, indicating a clear link between systemic inflammation and HF severity. **Conclusions**: The significant elevation of IL-1, IL-6, and TNF-α in HF patients highlights the pivotal role of inflammation in disease progression and severity, offering valuable insights into the underlying mechanisms that may inform future therapeutic strategies. By providing a comprehensive evaluation of these key pro-inflammatory cytokines in relation to LVEF, this study presents an integrated perspective on the inflammatory profile associated with HF.

## 1. Introduction

Heart failure (HF) is a major global public health problem, affecting approximately 64 million people worldwide and associated with high morbidity, mortality, and healthcare costs [1]. Despite substantial advancements in pharmacological and interventional therapies, the prevalence and impact of HF continue to rise, driven by aging populations and the increasing burden of risk factors such as hypertension, diabetes mellitus, and obesity [2,3]. HF is a progressive clinical syndrome characterized by structural and functional cardiac abnormalities that impair the heart’s ability to deliver adequate blood to meet the body’s metabolic demands. This leads to classic symptoms such as dyspnea, fatigue, and fluid retention, significantly diminishing patients’ quality of life and survival [4,5].

Traditionally, the pathophysiology of HF has been attributed primarily to hemodynamic and neurohormonal disturbances. However, accumulating evidence highlights a critical role for chronic systemic inflammation in the initiation and progression of HF [6]. Persistent inflammatory activation contributes to adverse cardiac remodeling, interstitial fibrosis, endothelial dysfunction, and ultimately to worsening myocardial function [7].

Cytokines such as interleukin-1 (IL-1), interleukin-6 (IL-6), and tumor necrosis factor-alpha (TNF-α) have been identified as key mediators in this inflammatory cascade [8]. These molecules interact in complex signaling networks, amplifying local and systemic inflammatory responses that exacerbate cardiac dysfunction and accelerate disease progression. Understanding the individual and collective contributions of these cytokines is essential for decoding the inflammatory mechanisms underpinning HF and identifying potential diagnostic and therapeutic targets [9].

Interleukin-1 (IL-1) is a potent pro-inflammatory cytokine that plays a central role in initiating and sustaining inflammatory responses within the myocardium. It impairs myocardial contractility, promotes cardiomyocyte apoptosis, and contributes to extracellular matrix remodeling, ultimately resulting in ventricular dilation and systolic dysfunction [10]. Elevated IL-1 levels have been consistently associated with reduced left ventricular ejection fraction (LVEF), higher NYHA functional class, and adverse clinical outcomes in HF patients [11].

Interleukin-6 (IL-6) is a multifunctional cytokine that further perpetuates inflammatory and fibrotic processes. IL-6 stimulates fibroblast proliferation, enhances collagen deposition, and promotes myocardial hypertrophy, thereby exacerbating ventricular stiffness and impairing diastolic function [12]. Clinically, increased IL-6 levels have been correlated with higher mortality rates, advanced HF symptoms, and greater degrees of ventricular dysfunction [13].

Tumor necrosis factor-alpha (TNF-α) is another critical mediator that exerts a wide range of deleterious effects on the myocardium. TNF-α induces cardiomyocyte apoptosis, alters β-adrenergic signaling, impairs calcium homeostasis, and promotes systemic catabolic states contributing to muscle wasting and cachexia [14]. Elevated TNF-α levels are strongly linked to worse clinical outcomes, including reduced LVEF, increased hospitalization rates, and higher mortality [15].

Given their central roles in the inflammatory pathophysiology of HF and their documented associations with disease severity and prognosis, IL-1, IL-6, and TNF-α represent promising biomarkers for both diagnostic and prognostic purposes. Moreover, these cytokines may serve as potential targets for novel anti-inflammatory therapeutic strategies, which could complement existing heart failure treatments and improve patient outcomes [16].

Despite growing evidence, previous studies often focused on individual cytokines and lacked a comprehensive, comparative analysis across diverse patient populations. As a result, the precise relationships between multiple inflammatory markers and cardiac function parameters remain insufficiently understood and require further elucidation.

In this context, the present study was designed to evaluate serum levels of IL-1, IL-6, and TNF-α in patients with HF compared to the control group, to investigate their associations with LVEF. This integrated assessment of multiple key pro-inflammatory cytokines within a rigorously controlled case–control design provides a comprehensive view of the inflammatory landscape in HF. By clarifying these relationships, the study highlights the potential utility of these cytokines as biomarkers of disease severity and progression.

## 2. Materials and Methods

### 2.1. Study Design

This case–control study was conducted at Madinah Cardiac Center (MCC), a tertiary care hospital in Al-Madinah Al-Munawara, Saudi Arabia, between October 2024 and April 2025. The study protocol was approved by the Institutional Review Board of MCC (Approval code: H-03-M-143; Approval date: 14 October 2023). All participants provided written informed consent, and the study was conducted following the Declaration of Helsinki.

### 2.2. Study Population

A total of 126 participants were included: 61 patients diagnosed with HF and 65 age-, sex-, and BMI-matched control subjects. All HF patients were recruited from the outpatient cardiology clinic at MCC and were clinically stable at the time of enrollment, defined as the absence of hospital admissions, emergency visits, or documented episodes of acute decompensated heart failure within the preceding three months, ensuring that inflammatory marker measurements reflect stable, chronic HF rather than acute disease exacerbation. The required sample size was estimated using G*Power software (version 3.1.9.7; Heinrich-Heine-Universität Düsseldorf, Düsseldorf, Germany; http://www.gpower.hhu.de, accessed on 25 May 2025). The sample size was based on prior literature reporting significant differences in cytokine levels between HF and control groups and was sufficient to detect medium effect sizes with 80% power at a significance level (α) of 0.05 [17]. The analysis indicated that at least 45 participants per group would be necessary. Accordingly, our final sample of 61 patients and 65 controls was sufficient to meet and exceed these requirements, ensuring adequate power for group comparisons and correlation analyses. Eligible HF patients were adults (≥18 years) with a confirmed diagnosis of HF (NYHA class II–IV) based on clinical assessment and echocardiographic evidence. Only patients with a left ventricular ejection fraction (LVEF) below 50% (including heart failure with reduced ejection (HFrEF) ≤ 40% and heart failure with mildly reduced ejection (HFmrEF, 40–50%) were included. Control participants were individuals without a history of HF, matched to the patient group to reduce potential confounding. Exclusion criteria for all participants included malignancies, thyroid disorders, advanced chronic kidney disease (stage 4 or 5), liver disease, hematological disorders, congenital heart disease, active infections, autoimmune diseases, recent surgeries, or other inflammatory conditions affecting cytokine levels. Patients using immunomodulatory drugs and pregnant individuals were also excluded to minimize confounding. The etiology of HF in our cohort was ischemic in 37 patients (60.7%) and non-ischemic in 24 patients (39.3%), with the latter including idiopathic dilated cardiomyopathy, hypertensive heart disease, and valvular heart disease. Most patients were receiving guideline-directed medical therapy (GDMT) following international recommendations: 54 patients (88.5%) were on angiotensin-converting enzyme inhibitors, angiotensin receptor blockers, or angiotensin receptor–neprilysin inhibitors (ACEI/ARB/ARNI); 50 patients (82.0%) were on beta-blockers; 42 patients (68.9%) were on mineralocorticoid receptor antagonists; 33 patients (54.1%) were on sodium–glucose cotransporter 2 (SGLT2) inhibitors; and 48 patients (78.7%) were on loop diuretics. Additionally, 38 patients (62.3%) were on statin therapy for dyslipidemia and cardiovascular risk reduction, reflecting the high prevalence of comorbidities in this group. Comorbidities included diabetes mellitus in 32 patients (52.4%), hypertension in 30 patients (49.2%), and obesity (BMI ≥ 30 kg/m^2^) in 16 patients (26.2%). Regarding disease severity, 38 patients (62.3%) had HFrEF and 23 patients (37.7%) had HFmrEF. According to the NYHA classification, 19 patients (31.1%) were class II, 34 patients (55.7%) were class III, and 8 patients (13.1%) were class IV.

### 2.3. Data Collection and Measurements

Demographic and clinical data were obtained through structured interviews and medical records, including age, sex, marital status, occupation, residency, and detailed cardiac history. Physical assessments included height, weight, and BMI calculation, as well as cardiac examination. Electrocardiography (ECG) was performed to evaluate heart rate, rhythm, and electrical activity, while echocardiography assessed cardiac structure and function, with a focus on LVEF.

Laboratory evaluations comprised a complete blood count (CBC) (including RBC, WBC, hemoglobin, hematocrit, MCV, MCH), lipid profile (total cholesterol, LDL-C, HDL-C, triglycerides), and cardiac biomarker troponin I. Serum levels of IL-1, IL-6, and TNF-α were quantified using enzyme-linked immunosorbent assays (ELISA). IL-1 and IL-6 were measured using the Abcam plc kits; ab100562 and ab100573, respectively (Abcam, Cambridge, UK). While TNF-α was measured using the Quantikine^®^ ELISA kit (R&D Systems Inc., Minneapolis, MN, USA). The assays involved specific antibody-coated microplates, incubation with serum samples, and detection using biotinylated antibodies followed by streptavidin–horseradish peroxidase conjugate. The resulting colorimetric reaction was read at 450 nm, and concentrations were interpolated from standard curves.

### 2.4. Statistical Analysis

Data analysis was performed using SPSS software (version 21.0; IBM Corp., Armonk, NY, USA). Continuous variables were expressed as mean ± standard error (SE) and compared using Student’s *t*-test. Pearson’s correlation was used to assess associations between cytokine levels and LVEF for normally distributed data, while Spearman’s correlation was applied otherwise. Multivariate logistic regression analysis was conducted to identify independent predictors of HF. A two-sided *p*-value < 0.05 was considered statistically significant.

## 3. Results

### 3.1. Demographic Data

Table 1 provides a detailed comparison of demographic characteristics and major cardiovascular risk factors between heart failure patients and controls. Factors analyzed included age, sex, smoking status, diabetes mellitus (DM), hypertension (HTN), and body mass index (BMI). No statistically significant differences were observed between groups in the prevalence of DM (52.4% vs. 58.5%; *p* = 0.27), HTN (49.2% vs. 47.7%; *p* = 0.23), smoking (25.6% vs. 30.8%; *p* = 0.25), or obesity (BMI > 30; 26.2% vs. 29.2%; *p* = 0.32). The proportion of participants aged ≥ 45 years and the distribution of male sex were also comparable between patients and controls (*p* = 0.36 and *p* = 0.17, respectively). Importantly, the inclusion of controls with similar rates of DM, HTN, and other common cardiovascular risk factors reduces potential confounding and allows for a more accurate assessment of the independent association between inflammatory cytokine levels and heart failure. These findings confirm that the two groups were well-matched, supporting the robustness of the subsequent analyses. Within the heart failure cohort, ischemic etiology was predominant, accounting for 60.7% of cases, while 39.3% were of non-ischemic origin, including idiopathic dilated cardiomyopathy, hypertensive heart disease, and valvular heart disease. Regarding left ventricular systolic function, 62.3% of HF patients had HFrEF (LVEF ≤ 40%), and 37.7% had HFmrEF (LVEF 41–49%). Functional capacity based on the NYHA classification showed that 31.1% were in class II, 55.7% in class III, and 13.1% in class IV, reflecting a predominance of moderate-to-severe functional limitation. These severity profiles, combined with the high prevalence of comorbid conditions, provide important clinical context for interpreting the observed elevations in inflammatory cytokines.

### 3.2. Clinical Characteristics of HF Patients and Control Groups

The key clinical characteristics were summarized in Table 2, including blood pressure, lipid profile parameters, HbA1c, and troponin I levels, in heart failure patients compared to controls. Diastolic blood pressure was significantly lower in HF patients (*p* < 0.001), reflecting reduced peripheral resistance and impaired cardiac output commonly observed in advanced heart failure. HbA1c levels were comparable between groups (*p* = 0.38), indicating similar glycemic control. HDL cholesterol levels were lower in HF patients, although this difference did not reach statistical significance (*p* = 0.09). In contrast, LDL cholesterol was significantly higher among HF patients (*p* < 0.001), emphasizing its potential role in cardiovascular disease progression. Triglyceride levels were mild but significantly elevated in HF patients compared to controls (*p* = 0.036), suggesting an association with altered lipid metabolism. Additionally, troponin I levels were elevated in HF patients, supporting the presence of ongoing subclinical myocardial injury. These findings collectively highlight the metabolic and hemodynamic alterations characteristic of heart failure.

### 3.3. Inflammatory Cytokine Levels in Patients and Control Groups

**Figure 1:**
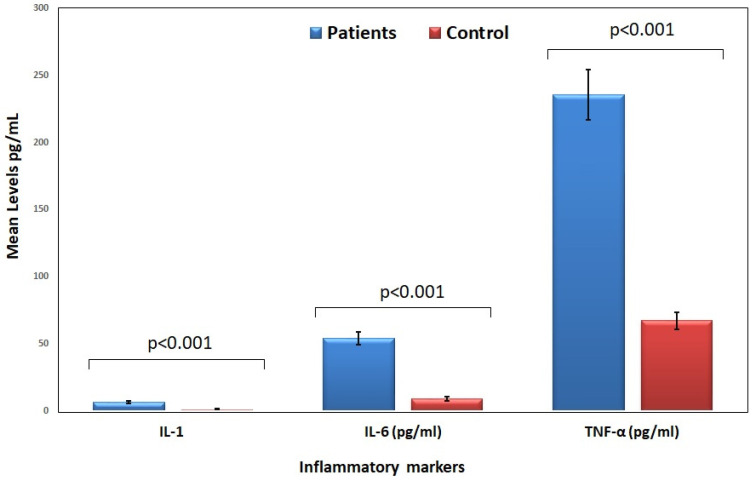
Inflammatory cytokine levels in patients and control groups.

Table 3 presents a comparison of key inflammatory cytokine levels (IL-1, IL-6, and TNF-α) between the patient group and the control groups. The mean levels and standard errors (SE) are provided along with statistical significance (*p*-value). This analysis helps determine the extent of systemic inflammation in HF patients compared to controls. The inflammatory cytokine levels (IL-1, IL-6, and TNF-α) are significantly higher in heart failure patients compared to the control group (*p* < 0.001 for all markers). This suggests a strong association between inflammation and heart failure, supporting the hypothesis that elevated inflammatory markers contribute to disease severity and progression. Figure 1 compares the mean levels of inflammatory markers in HF patients and controls. The bar chart highlights the significantly higher levels of IL-1, IL-6, and TNF-α in HF patients, reinforcing their role in systemic inflammation and disease progression.

Multivariate logistic regression analysis was conducted to identify independent predictors of heart failure (Table 4). The results revealed that higher serum levels of IL-1 (OR = 2.35; 95% CI: 1.45–3.81; *p* < 0.001), IL-6 (OR = 1.87; 95% CI: 1.12–3.14; *p* < 0.001), and TNF-α (OR = 2.92; 95% CI: 1.76–4.89; *p* < 0.001) were all significantly associated with increased odds of HF. Additionally, elevated LDL levels (OR = 1.48; 95% CI: 1.03–2.12; *p* = 0.034) also emerged as a significant risk factor. Interestingly, diastolic blood pressure was inversely associated with HF risk (OR = 0.82; 95% CI: 0.69–0.98; *p* = 0.026), suggesting that lower diastolic pressure may reflect advanced hemodynamic compromise in HF patients. These findings underscore the independent contribution of systemic inflammation and lipid dysregulation to heart failure pathogenesis.

**Figure 2:**
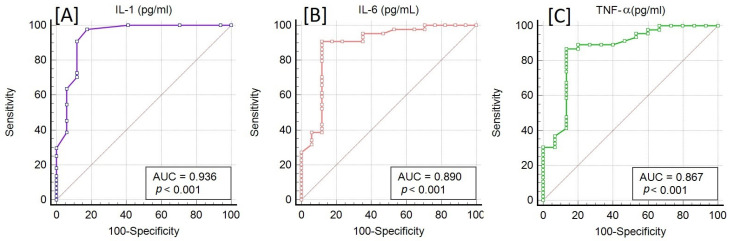
Receiver operating characteristic (ROC) curves for IL-1 (**A**), IL-6 (**B**), and TNF-α (**C**) in distinguishing HF patients from controls.

The diagnostic performance of IL-1, IL-6, and TNF-α in distinguishing HF patients from control subjects was assessed using Receiver operating characteristic (ROC) curve analysis (Figure 2), revealing a robust association between elevated cytokine levels and the presence of HF. Among the three markers, IL-1 demonstrated the highest discriminative ability, with an area under the curve (AUC) of 0.936 (*p* < 0.001), indicating excellent sensitivity and specificity. This suggests that IL-1 could serve as a particularly strong indicator of the inflammatory response in HF pathophysiology. IL-6 and TNF-α also exhibited strong diagnostic potential, with AUCs of 0.890 and 0.867, respectively (both *p* < 0.001), further supporting the role of systemic inflammation in HF development and progression. The consistent and statistically significant AUC values for all three cytokines suggest that inflammatory mediators are not only elevated in HF but may also serve as useful non-invasive biomarkers for early detection, stratification of disease severity, or monitoring of therapeutic responses. Importantly, the high sensitivity observed—particularly in IL-1—underscores the utility of this cytokine in identifying affected individuals even at earlier or subclinical stages.

### 3.4. Correlation Between Inflammatory Cytokine Markers and LV Ejection in HF Patients

**Figure 3:**
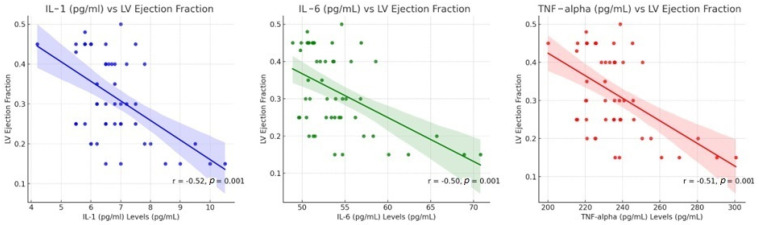
Correlation between inflammatory cytokine markers and LV ejection in HF patients.

Figure 3 illustrates the relationship between inflammatory markers (IL-1, IL-6, and TNF-α) and left ventricular (LV) ejection fraction. The scatter plots show a strong negative correlation, indicating that as cytokine levels increase, LV ejection fraction decreases, suggesting a direct link between inflammation and worsening cardiac function. This figure presents three scatter plots displaying the relationship between inflammatory markers (IL-1, IL-6, and TNF-α) and left ventricular (LV) ejection fraction. A strong negative correlation (r = −0.52, *p* < 0.001) indicates that higher IL-1 levels are associated with a lower ejection fraction. IL-6 levels are also negatively correlated with LV ejection fraction (r = −0.50, *p* < 0.001). Similarly, TNF-α shows a strong inverse relationship (r = −0.51, *p* < 0.001). Each inflammatory marker (IL-1, IL-6, and TNF-α) is strongly associated with decreased LV ejection fraction. This supports the hypothesis that inflammation exacerbates cardiac dysfunction in HF patients.

## 4. Discussion

This study concentrates on the fundamental impact of inflammation in HF pathophysiology. increased IL-1, IL-6, and TNF-α levels were calculated and noted in HF patients, evaluating the association of their levels with the severity of the disease [18,19]. The strong correlation between inflammatory markers and LVEF suggests their possible role as prognostic indicators [20,21]. In addition, comorbidities such as DM and HTN were more prevalent in HF patients, reinforcing their contribution to disease progression [22]. In our cohort, systolic blood pressure did not significantly differ between HF patients and controls, consistent with prior studies in stable HF populations receiving optimized medical therapy, where pharmacologic management may maintain systolic pressures within the normal range despite impaired cardiac function [23]. Conversely, diastolic pressure was significantly lower in HF patients, in agreement with reports linking reduced diastolic blood pressure to decreased vascular compliance and advanced disease stages [24]. Additionally, HF patients had higher LDL cholesterol, but similar HbA1c levels compared to controls, suggesting concomitant dyslipidemia and hemodynamic compromise rather than purely inflammatory effects. Notably, when LDL cholesterol and diastolic BP were included in our multivariate logistic regression model alongside IL-1, IL-6, and TNF-α, all three cytokines remained independent predictors of HF. This indicates that systemic inflammation contributes to HF pathophysiology beyond the influence of lipid levels and blood pressure, highlighting its role as a distinct pathogenic mechanism.

Our findings are consistent with prior research that has identified inflammation as a central driver of myocardial remodeling and functional deterioration in HF. Elevated TNF-α levels have been linked to cardiomyocyte apoptosis, mitochondrial dysfunction, and myocardial fibrosis, all of which contribute to progressive systolic dysfunction and adverse clinical outcomes [25]. Elevated levels of TNF-α have been linked to cardiomyocyte apoptosis, mitochondrial dysfunction, and myocardial fibrosis, which lead to deterioration of heart function [26,27]. Similarly, IL-6 has been shown to promote progression of ventricular remodeling, fibrosis, and hypertrophy, ongoing worsening the clinical course of HF [28]. IL-1, which is known for its function in systemic inflammation, has been implicated in endothelial dysfunction and impaired myocardial contractility [29], emphasizing the potential of targeting these pathways for treatments. In our study, ischemic etiology accounted for 60.7% of HF cases, while 39.3% were non-ischemic, including idiopathic dilated cardiomyopathy, hypertensive heart disease, and valvular heart disease. Most patients were on comprehensive GDMT, with 88.5% receiving ACEI/ARB/ARNI, 82.0% beta-blockers, 68.9% mineralocorticoid receptor antagonists, 54.1% SGLT2 inhibitors, and 78.7% loop diuretics. The widespread use of these evidence-based therapies may have influenced the absolute levels of inflammatory cytokines, potentially attenuating systemic inflammation compared with untreated or acutely decompensated populations. Nevertheless, the significant differences observed between HF patients and matched controls indicate that substantial residual inflammation persists despite optimized medical treatment [30]. Furthermore, most of our cohort had moderate-to-severe functional limitations, with over half in NYHA class III or IV, and nearly two-thirds exhibiting HFrEF. This severity profile, combined with the high prevalence of comorbidities such as diabetes mellitus, hypertension, and obesity, likely contributes to the elevated inflammatory marker levels observed.

The strong inverse relationship observed between inflammatory cytokines and LVEF in our cohort suggests that chronic systemic inflammation directly contributes to cardiac functional decline. These results support the utility of IL-1, IL-6, and TNF-α as potential biomarkers to identify patients at higher risk for adverse outcomes. Routine measurement of these markers in clinical practice could help guide risk stratification and inform more intensive monitoring or therapeutic approaches in high-risk individuals [31]. This finding confirms the possible usefulness of IL-1, IL-6, and TNF-α as biomarkers for HF severity and prognosis [32]. Their measurement in clinical practice may help identify high-risk patients who would benefit from frequent assessment and more invasive therapeutic plans [33].

The significant correlation between inflammatory markers and HF brings attention to a key factor influencing treatment decisions. ongoing treatment methods for HF primarily focus on neurohormonal blockades, including angiotensin-converting enzyme inhibitors (ACEIs), beta-blockers, and mineralocorticoid receptor antagonists. However, recent research has suggested that anti-inflammatory therapies could be used for additional benefits for HF patients [33]. For instance, IL-1 receptor antagonists, TNF inhibitors, and IL-6 blockers have been investigated as possible therapeutic strategies to decline systemic inflammation and enhance cardiac function [34]. While some early trials have shown promise, larger randomized controlled studies are needed to confirm their efficacy and safety in HF patients [35].

Another important aspect highlighted by our findings is the potential role of inflammation as a therapeutic target in HF prevention. Patients with risk factors such as HTN, DM, and obesity may experience low-grade persistent inflammation that will contribute to early myocardial dysfunction [36]. Detecting and assessing inflammation at earlier stages of HF progression may postpone or even prevent the onset of symptomatic disease. Further studies should explore whether lifestyle interventions, such as diet and exercise, in addition to anti-inflammatory medications, could contribute to durable therapeutic effects in reducing HF incidence and progression [37]. Furthermore, the concurrent assessment of these three cytokines provides a more integrated view of the inflammatory signature associated with HF. Rather than evaluating markers in isolation, combining their diagnostic potential may enhance clinical decision-making and improve risk stratification models. Taken together, the ROC curve analyses reinforce the clinical relevance of inflammation in HF and support the exploration of anti-inflammatory strategies as potential therapeutic approaches.

Despite the robust associations observed, several limitations must be acknowledged. First, the cross-sectional design of our study precludes the establishment of causality between elevated cytokine levels and HF progression. Longitudinal studies are warranted to determine whether increases in inflammatory markers precede HF development or reflect ongoing disease severity. Second, although our patient and control groups were well-matched for age, sex, and BMI, other potential confounders, including genetic predisposition, medication use, and unmeasured comorbidities, could have influenced cytokine levels. Although all HF patients were clinically stable at enrollment and we excluded comorbid conditions known to affect cytokine levels, the absence of stratified analyses by HF etiology, medication regimen, or other clinical variables means that residual confounding cannot be entirely excluded. It is therefore possible that part of the observed cytokine elevation reflects these factors rather than HF severity alone. Future research should include larger, more diverse populations and comprehensive data collection to validate and expand upon these findings. Moreover, the cross-sectional design limits our ability to assess dynamic changes in cytokine levels over time or in response to treatment. Although cardiac function was assessed using LVEF and troponin I, NT-proBNP was not measured due to resource limitations, which may have provided additional complementary prognostic information.

In general, this study reinforces the increasing body of evidence that inflammation plays a crucial role in HF pathophysiology. Elevated levels of IL-1, IL-6, and TNF-α are strongly associated with HF severity and decline in cardiac function. These findings emphasize the importance of further exploring inflammation-targeted therapies as potential treatment options for HF patients.

## 5. Conclusions

This study demonstrates that IL-1, IL-6, and TNF-α are significantly elevated in heart failure patients and strongly correlate with reduced LVEF, indicating their association with disease severity. The combined assessment of these cytokines, rather than focusing on single markers, provides a more comprehensive characterization of the inflammatory milieu in HF within a rigorously controlled case–control design. Importantly, substantial systemic inflammation was observed despite optimized GDMT and in a predominantly advanced HF cohort. While these findings highlight the potential of cytokine profiling to improve HF diagnosis and risk stratification, their therapeutic targeting should be regarded as a hypothesis-generating concept. Future studies should validate these biomarkers in larger and more diverse populations and investigate whether cytokine-modulating strategies can favorably influence HF outcomes.

## Figures and Tables

**Table 1:** General characteristics of study subjects.

Factor	Category	Patients (%)	Controls (%)	*p*-Value
Age ≥ 45	Yes	83.6%	78.5%	0.36
	No	16.4%	21.5%	
DM	Yes	52.4%	58.5%	0.27
	No	47.6%	41.5%	
HTN	Yes	49.2%	47.7%	0.23
	No	50.8%	53.3%	
Male gender	Yes	67.2%	70.8%	0.17
	No	32.8%	29.2%	
Smoking	Yes	25.6%	30.8%	0.25
	No	74.4%	69.2%	
BMI > 30	Yes	26.2%	29.2%	0.32
	No	73.8%	70.8%	

DM: diabetes mellitus; HTN: hypertension; BMI: body mass index.

**Table 2:** The clinical characteristics of HF patients and control groups.

Variable	Patients (Mean ± SE)	Control (Mean ± SE)	*p*-Value
Systolic BP (mmHg)	116.94 ± 13.61	118.04 ± 11.98	0.915
Diastolic BP (mmHg) HbA1c (%)	68.73 ± 8.80 7.59 ± 2.71	77.95 ± 10.70 6.89 ± 2.15	<0.001 0.38
HDL (mmol/L)	1.04 ± 0.36	1.17 ± 0.11	0.09
LDL (mmol/L)	2.33 ± 0.72	1.84 ± 0.65	<0.001
Triglycerides (mmol/L) Troponin I (ng/mL)	1.39 ± 0.71 0.15 ± 0.02	1.27 ± 0.93 -	0.036 N/A

HbA1c: glycated hemoglobin A1c; HDL: high-density lipoprotein cholesterol; LDL: low-density lipoprotein cholesterol: N/A: nonapplicable.

**Table 3:** Inflammatory cytokine levels in patients and control groups.

Variable	Patients (Mean ± SE)	Control (Mean ± SE)	*p*-Value
IL-1 (pg/mL)	6.77 ± 1.17	1.27 ± 0.42	<0.001
IL-6 (pg/mL)	54.12 ± 4.64	9.29 ± 1.72	<0.001
TNF-α (pg/mL)	235.56 ± 18.88	67.37 ± 6.28	<0.001

IL-1: interleukin-1; IL-6: interleukin-6; TNF-α: tumor necrosis factor-alpha.

**Table 4:** Multivariate logistic regression analysis identifying independent predictors of heart failure.

Variable	Odds Ratio (OR)	95% CI	*p*-Value
IL-1 (pg/mL)	2.35	1.45–3.81	<0.001
IL-6 (pg/mL)	1.87	1.12–3.14	<0.001
TNF-α (pg/mL)	2.92	1.76–4.89	<0.001
LDL (mmol/L)	1.48	1.03–2.12	0.034
Diastolic BP (mmHg)	0.82	0.69–0.98	0.026

IL-1: interleukin-1; IL-6: interleukin-6; TNF-α: tumor necrosis factor-alpha; LDL: low-density lipoprotein cholesterol; BP: blood pressure.

## Data Availability

Data sharing is applicable to this article.
